# Increased Ca buffering underpins remodelling of Ca^2+^ handling in old sheep atrial myocytes

**DOI:** 10.1113/JP274053

**Published:** 2017-09-11

**Authors:** Jessica D. Clarke, Jessica L. Caldwell, Charles M. Pearman, David A. Eisner, Andrew W. Trafford, Katharine M. Dibb

**Affiliations:** ^1^ Unit of Cardiac Physiology, Manchester Academic Health Sciences Centre, Central Manchester Foundation Trust, 3.14 Core Technology Facility University of Manchester Manchester UK

**Keywords:** age, atria, buffering, calcium

## Abstract

**Key points:**

- Ageing is associated with an increased risk of cardiovascular disease and arrhythmias, with the most common arrhythmia being found in the atria of the heart.
- Little is known about how the normal atria of the heart remodel with age and thus why dysfunction might occur.
- We report alterations to the atrial systolic Ca^2+^ transient that have implications for the function of the atrial in the elderly.
- We describe a novel mechanism by which increased Ca buffering can account for changes to systolic Ca^2+^ in the old atria.
- The present study helps us to understand how the processes regulating atrial contraction are remodelled during ageing and provides a basis for future work aiming to understand why dysfunction develops.

**Abstract:**

Many cardiovascular diseases, including those affecting the atria, are associated with advancing age. Arrhythmias, including those in the atria, can arise as a result of electrical remodelling or alterations in Ca^2+^ homeostasis. In the atria, age‐associated changes in the action potential have been documented. However, little is known about remodelling of intracellular Ca^2+^ homeostasis in the healthy aged atria. Using single atrial myocytes from young and old Welsh Mountain sheep, we show the free Ca^2+^ transient amplitude and rate of decay of systolic Ca^2+^ decrease with age, whereas sarcoplasmic reticulum (SR) Ca content increases. An increase in intracellular Ca buffering explains both the decrease in Ca^2+^ transient amplitude and decay kinetics in the absence of any change in sarcoendoplasmic reticulum calcium transport ATPase function. Ageing maintained the integrated Ca^2+^ influx via *I*_Ca‐L_ but decreased peak *I*_Ca‐L_. Decreased peak *I*_Ca‐L_ was found to be responsible for the age‐associated increase in SR Ca content but not the decrease in Ca^2+^ transient amplitude. Instead, decreased peak *I*_Ca‐L_ offsets increased SR load such that Ca^2+^ release from the SR was maintained during ageing. The results of the present study highlight a novel mechanism by which increased Ca buffering decreases systolic Ca^2+^ in old atria. Furthermore, for the first time, we have shown that SR Ca content is increased in old atrial myocytes.

## Key points

- Ageing is associated with an increased risk of cardiovascular disease and arrhythmias, with the most common arrhythmia being found in the atria of the heart.
- Little is known about how the normal atria of the heart remodel with age and thus why dysfunction might occur.
- We report alterations to the atrial systolic Ca^2+^ transient that have implications for the function of the atrial in the elderly.
- We describe a novel mechanism by which increased Ca buffering can account for changes to systolic Ca^2+^ in the old atria.
- The present study helps us to understand how the processes regulating atrial contraction are remodelled during ageing and provides a basis for future work aiming to understand why dysfunction develops.

**Abbreviations**
AF: atrial fibrillation
NCX: Na^+^/Ca^2+^ exchange
PLB: phospholamban
SERCA: sarcoendoplasmic reticulum calcium transport ATPase
PP: protein phosphatase
RyR: ryanodine receptor
SR: sarcoplasmic reticulum

## Introduction

Age is a major risk factor for cardiovascular disease and, as life expectancy continues to increase, so too does the prevalence of cardiovascular disease (Kirkwood, 2008; Lakatta, 2015). Despite the clinical and economic consequences of cardiovascular disease amongst the elderly, the mechanisms by which ageing causes heart disease remain poorly understood and underexplored.

Arrhythmias form a significant component of age‐associated cardiovascular disease and atrial fibrillation (AF) is the most commonly seen. AF has a greater probability of occurring when atrial myocytes have remodelled in response to diseases such as heart failure and previous episodes of AF, or as a consequence of the ageing process itself. Disease‐associated remodelling at the level of the cardiac myocyte includes alterations to excitation–contraction coupling contributing to contractile dysfunction (He *et al*. 2001; Lenaerts *et al*. 2009). Remodelling of excitation–contraction coupling also occurs in response to ageing and, although this is well described in ventricular myocytes (Dibb *et al*. 2004), it has been studied far less in the atria (Feridooni *et al*. 2015). Given the strong association between age and diseases of the atria, there is a pressing need to understand normal age‐associated remodelling in the atria because this is the substrate upon which dysfunction develops in the elderly.

Several facets of Ca^2+^ regulation have been shown to change with age. *I*_Ca‐L_ is decreased in old rabbits, dogs and humans (Cain *et al*. 1998; Dun *et al*. 2003; Wongcharoen *et al*. 2007; Gan *et al*. 2013; Xu *et al*. 2013; Herraiz *et al*. 2015). Atrial Ca^2+^ transient amplitude and sarcoplasmic reticulum (SR) Ca content are decreased in aged patients (Herraiz *et al*. 2015). However, because human studies are hampered by confounding factors from underlying disease, it remains to be determined how these aspects of Ca^2+^ handling are remodelled during ageing in the absence of confounding diseases. We have therefore used a sheep model of ageing aiming: (i) to define how Ca^2+^ handling is altered and (ii) to determine the mechanisms that are responsible for altered Ca^2+^ homeostasis in the old atria.

The sheep atrium was chosen for the present study because of its similarity to human atria (e.g. a similar complement of ionic currents and the presence of an extensive atrial t‐tubule network) (Richards *et al*. 2011). Furthermore, sheep, similar to humans, can spontaneously develop AF (Moresco *et al*. 2001) and thus may be more comparable to aged humans than rodents, which do not spontaneously develop AF. Although much previous work has focused on the right atrium, AF is normally initiated in the left atrium (Haissaguerre *et al*. 1998). This not only commonly occurs in the pulmonary vein sleeves, but may also arise from the left atrial appendage (Di *et al*. 2010). We have therefore used myocytes from the left atrial appendage to assess how intracellular Ca^2+^ handling changes with age.

In the present study, we report a novel mechanism for aberrant Ca^2+^ handling in the aged atria. We show that, in old sheep, both the amplitude and rate constant of decay of the Ca^2+^ transient decrease. These effects appear to result largely from an age‐associated increase in Ca^2+^ buffering.

## Methods

### Animal model

Animals in the present study were considered to represent healthy ageing. Young adult (∼18 months) and old (>8 years) female Welsh Mountain sheep deemed to be in their last quintile of life by clinical examination (Pope, 1934) without significant observable underlying disease were used. Animals demonstrating minor ailments such as non‐malignant dermatological and ocular conditions were included in the study, and these pathologies might be expected to occur more frequently in older animals (Rockwood *et al*. 2017). In a separate group of animals, we have previously shown that, although the heart rate increases with age, spontaneous AF or left ventricular hypertrophy do not occur (Horn *et al*. 2012, 2016)

### Ethical approval

All procedures comply with The UK Animals (Scientific Procedures) Act 1986 and EU directive 2010/63. The ARRIVE guidelines were followed for reporting the use of animals in scientific experiments. Local ethical approval was granted by The University of Manchester Animal Welfare Ethical and Review Board.

### Myocyte isolation

Single myocytes were isolated using an enzymatic digestion technique, as described previously (Dibb *et al*. 2009; Voigt *et al*. 2015). Animals were killed by i.v. injection of pentobarbitone (200 mg kg^−1^) mixed with heparin (10, 000 IU) and then the heart was rapidly removed and rinsed in Ca^2+^ free solution containing (mm): 134 NaCl, 11 glucose, 10 Hepes, 10 2,3‐butanedione monoxime, 4 KCl, 1.2 MgSO_4_, 1.2 NaH_2_PO_4_ and 0.5 mg ml^−1^ bovine serum albumin (pH 7.34 with NaOH). The atria were separated from the ventricles, cannulated via the left superior atrial artery and perfused at 37°C with the Ca^2+^ free solution for 10 min. Collagenase type II (0.55 mg ml^−1^; Worthington, Reading, UK) and protease type XIV (0.06 mg ml^−1^; Sigma, Poole, UK) were added to the superfusate and the heart digested for ∼12 min. The perfusate was switched to a taurine containing solution (in mm): 113 NaCl, 50 taurine, 11 glucose, 10 Hepes, 10 2,3‐butanedione monoxime, 4 KCl, 1.2 MgSO_4_, 1.2 NaH_2_PO_4_, 0.1 CaCl_2_ and 0.5 mg ml^−1^ BSA (pH 7.34 with NaOH) for a further 20 min. The left auricle was excised and myocytes were dispersed by chopping, followed by trituration. Prior to experimentation, cells were stored in a solution containing (mm): 140 NaCl, 10 glucose, 10 Hepes, 4 KCl, 1.8 CaCl_2_, 1 MgCl_2_ and 2 probenecid (pH 7.34 with NaOH) at room temperature.

### Measurement of intracellular Ca^2+^ and cellular electrophysiology

Atrial myocytes were loaded with Fluo‐5F AM to measure free intracellular Ca^2+^ [Ca^2+^]_i_ (Molecular Probes, Eugene, OR, USA). Cells were loaded at 5 μmol l^−1^ for 8 min and allowed to de‐esterify for at least 45 min prior to experimentation. Fluorescence was excited at 488 nm, emitted light captured >515 nm and intracellular Ca^2+^ calculated using:

(1) [Ca2+]i=KdF−F min F max −F

where, at a single wavelength, *K*_d_ is the dissociation constant of Fluo‐5F at 37°C (1035 nmol l^−1^) (Loughrey *et al*. 2003), *F* is fluorescence, *F*_min_ is fluorescence in the absence of Ca^2+^ (zero for Fluo‐5F) (Cheng *et al*. 1993) and *F*_max_ is the Ca^2+^ saturated fluorescence obtained at the end of the experiment (Trafford *et al*. 1999).

Electrophysiological experiments were performed simultaneously with [Ca^2+^]_i_ measurements (Dibb *et al*. 2005; Dibb *et al*. 2007). Cell dimensions were determined prior to patch clamp. Voltage clamp control was achieved using the perforated patch clamp technique with amphotericin‐B (240 μg ml^−1^) in the pipette solution (Trafford *et al*. 1999). The switch clamp facility (frequency 3–5 kHz) of the Axoclamp‐2B voltage clamp amplifier (Axon Instruments, Foster City, CA, USA) was used to overcome the access resistance (39.0 ± 3.1 MΩ) of the perforated patch. Any contaminating K^+^ and Cl^–^ currents were blocked by addition of (in mm): 5 4‐aminopyridine, 0.1 BaCl_2_ and 0.1 DIDS. Micropipettes (2–3 MΩ) were filled with (in mm): 125 KCH_3_O_3_S, 20 KCl, 10 NaCl, 10 Hepes and 5 MgCl_2_ and then titrated to pH 7.2 with KOH. *I*_Ca‐L_ was elicited by a 100 ms voltage step from a holding potential of −40 mV. We predict a liquid junction potential of −8.2 mV for all data, which has not been corrected for. To determine whether increasing Ca buffering in young atrial myocytes could produce an ‘old’ systolic Ca^2+^ transient, young atrial myocytes were incubated with 20 μm EGTA for 10 min. All experiments were performed at 37°C within 12 h of myocyte isolation.

### Sarcolemmal fluxes, SR Ca content and intracellular Ca buffering

Sarcolemmal fluxes [*I*_Ca‐L_ and Na^+^/Ca^2+^ exchange (NCX)] were quantitatively measured, as described previously (Varro *et al*. 1993; Trafford *et al*. 2001). *I*_Ca‐L_ was measured from the peak inward current to the steady‐state current at the end of the test pulse under control conditions. SR Ca content was assessed by releasing Ca^2+^ from the SR on application of 10 mmol l^−1^ caffeine at –40 mV. The resultant NCX current was integrated and expressed relative to total cell volume obtained from capacitance measurements in each cell using a capacitance to volume ratio of 4.8 pF pl^–1^ calculated in a separate set of experiments (described below). The integral was also corrected for Ca^2+^ removal by non‐electrogenic mechanisms (e.g. plasma membrane Ca²⁺ ATPase and the mitochondrial uniporter; see below).

Ca buffering was assessed as described previously (Trafford *et al*. 1999). During the application of caffeine, the corrected integral of the NCX current gives a measure of the total intracellular Ca (free Ca^2+^ + Ca bound to intracellular buffers), whereas a simultaneous record of the intracellular free Ca^2+^ concentration is obtained from Fluo‐5F. The relationship between these parameters gives the buffering power of the cell. Data points were fit by the hyperbolic equation:

(2) Ca T=a+Bmax·[ Ca 2+]iKd+[ Ca 2+]i

where the Ca_T_ is total Ca, *a* is an offset because the relationship cannot be determined for values of [Ca^2+^]_i_ below the resting level, *B*_max_ is the maximum buffering capacity for Ca^2+^, [Ca^2+^]_i_ represents the free intracellular Ca^2+^ and *K*_d_ is the dissociation constant of the buffers. Ca buffer power was calculated from the *B*_max_ and *K*_d_ values obtained above using the equation (Díaz *et al*. 2001a):

(3) d( Ca T)d[ Ca 2+]i=Bmax·Kd[ Ca 2+]i+Kd2

The increase of Ca_T_ during the Ca^2+^ transient (∆[Ca]_Total_) was calculated from the resting and peak systolic [Ca^2+^]_i_ and the buffer properties. The amount of Ca^2+^ released from the SR was then calculated by subtracting the integral of the L‐type Ca^2+^ current from this value. The interaction between sarcolemmal Ca^2+^ fluxes and SR Ca^2+^ release were assessed by measuring both the gain of CICR and the fractional release of Ca^2+^ from the SR. Gain was calculated using the equation:

(4) ECcouplinggain=Δ[Ca] Total −∫I Ca −L Peak I Ca −L density

The fractional release of Ca^2+^ from the SR was calculated using eqn (5):

(5) f SR =Δ[Ca] Total −∫I Ca −L SR Ca content

### Measurement of capacitance to volume relationship in single atrial myocytes

The capacitance to volume relationship was quantified using an SP2 confocal microscope (Leica Microsystems, Wetzlar, Germany), as described previously (Walden *et al*. 2009; Satoh *et al*. 1996). Both young and old atrial myocytes were loaded with calcein‐AM (20 μmol l^−1^ for 20 min at room temperature). Cells were excited at a wavelength of 488 nm and emitted light collected >500 nm. Imaging was performed at an *xy* resolution of 310 nm and 310 nm separation between *z* stacks. Cells were subsequently patch clamped and capacitance measured to generate a capacitance–volume relationship. This relationship was not altered by age and we therefore used a combined value of 4.80 ± 0.18 pF pl^–1^.

### Correction for Ca^2+^ removal by pathways other than sarcoendoplasmic reticulum calcium transport ATPase (SERCA) and NCX

Correction factors were calculated in a further series of experiments as described previously (Varro *et al*. 1993; Dibb *et al*. 2004). Briefly, cells were electrically paced under field stimulation at 0.5 Hz using a pair of silver wires attached to a Digitimer DS2A (Digitimer Ltd, Welwyn Garden City, UK). SERCA was effectively disabled (with caffeine) and then both SERCA and NCX together were blocked by application of Ni^+^ and caffeine. The rates of decay of the caffeine‐evoked Ca^2+^ transient (*k*_caff_) and the caffeine‐evoked Ca^2+^ transient in the presence of 10 mmol l^−1^ Ni^+^ (*k*_Ni+caff_) were calculated by fitting single exponentials. Correction factors were calculated for each age group using eqn (6) and were found to be 1.14 ± 0.03 and 1.26 ± 0.05 for young and old atrial myocytes, respectively:

(6) CF =k caff k caff −k Ni + caff

These correction factors were used to calculate SR Ca content and Ca buffering.

### Western blotting

Following removal of the heart, left atrial appendage tissue was dissected from a subset of animals not used for cell isolation and stored in liquid nitrogen until use. Proteins were isolated as described previously (Díaz *et al*. 2004; Graham & Trafford, 2007; Caldwell *et al*. 2014; Clarke *et al*. 2015). In brief, samples were homogenized in radio immunoprecipitation assay (RIPA) buffer containing phosphatase inhibitors and the protein concentration was quantified using a DC Protein Assay kit (Bio‐Rad, Hercules, CA, USA). Samples were separated using denaturing SDS‐PAGE, transferred to nitrocellulose membrane and blocked using either Superblock or SeaBlock (Thermo Scientific, Waltham, MA, USA). SERCA, phospholamban (PLB), phosphorylated PLB (at Thr17 and Ser16), protein phosphatase (PP)1 and PP2a, ryanodine receptor (RyR), and phosphorylated RyR (at serine 2808 and serine 2814) immunodetection was performed using chemiluminescent substrate (Supersignal West Pico Chemiluminescent Substrate; Thermo Scientific) and captured digitally (Syngene, Cambridge, UK). Loading controls such as β‐actin or GAPDH have been found to be altered in disease or are unreliable and we therefore performed each blot in triplicate (Briston *et al*. 2011; Caldwell *et al*. 2014; Clarke *et al*. 2015). An internal protein standard, obtained from the left atrium of a single control sheep (internal control), was loaded on each blot and used to normalize protein levels to allow comparison between experiments. Mean data from three repeats for each western blot containing samples from seven to 10 animals per experimental group are presented. All secondary antibodies were HRP conjugates and were visualized using chemiluminescence (Syngene).

### Statistical analysis

Data are presented as the mean ± SEM (*n* animals and cells). Differences between young and old animals were determined using linear mixed modelling (SPSS, version 20; IBM Corp., Armonk, NY, USA) to account for multiple cellular data points from a single sheep. Thus, for statistical purposes, in the present study, *n* is the number of animals. The Kolmogorov–Smirnov or the Shapiro–Wilk statistic (SPSS, version 20) was used to test for normality of data distribution. Data were transformed appropriately (e.g. log_10_ transform) to achieve a normal distribution. In a small number of cases where data could not be normalized with an appropriate transformation, linear mixed modelling was performed if the frequency distribution of the data was symmetrical or *n* was sufficiently large (Ennos, 2000; Gelman & Hill, 2007).

## Results

### Ageing results in cellular hypertrophy

**Figure 1:**
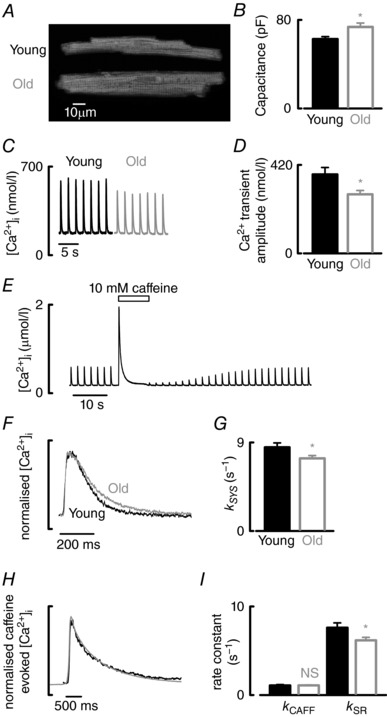
Ageing increases cell size and decreases Ca^2+^ transient amplitude and rate of decay of systolic Ca^2+^ in the atria *A*, confocal images of typical atrial myocytes from young and old sheep loaded with calcein‐AM. *B*, mean cellular capacitance of atrial myocytes from young and old animals (*n *= 40–53 cells, 16–20 animals). *C*, representative systolic Ca^2+^ transients from young and old atrial myocytes elicited under voltage clamp control. *D*, mean systolic Ca^2+^ transient amplitude calculated from data similar to (*C*) (*n *= 29–51 cells, 15–23 animals). *E*, typical experimental time course for Ca^2+^ including application of 10 mm caffeine. *F*, example normalized systolic Ca^2+^ transients. *G*, mean rate of decay of the systolic Ca^2+^ transient (*k*_SYS_) (*n *= 29–51 cells, 15–23 animals). *H*, normalized caffeine‐evoked Ca^2+^ transients. *I*, mean data summarizing SERCA (*k*_SR_) and sarcolemmal (*k*_CAFF_)‐dependent rates of Ca^2+^ removal (*n *= 22–38 cells, 11–20 animals). ^*^ *P *< 0.05.

Typical calcein‐AM stained atrial myocytes from young and old hearts are shown in Fig. 1 *A*. Both cell length and width increased with age by 7 ± 2% and 17.2 ± 3%, respectively (Table 1). Consistent with increased cell size, capacitance (indicative of the amount of surface membrane) (Fig. 1 *B*) increased with age by 17 ± 7% (Table 1). Simultaneous measurements of cell volume (obtained from confocal *z* sections through the entire calcein loaded cell) and capacitance (patch clamp) were performed in young and old cells. Volume increased by 45% with age (13.5 ± 1.4 *vs*. 19.7 ± 1.7 pl, *P *< 0.05, *n *= 17–19 cells from 5–7 animals). Increased volume was accompanied by an equivalent increase in cellular capacitance such that the ratio of capacitance to volume was unchanged during ageing (5.06 ± 0.17 *vs*. 4.51 ± 0.32 pF pl^–1^, not significant, *n *= 17–19 cells and 5–7 animals). A mean value of capacitance for a given cell volume was therefore generated (4.80 ± 0.18 pF pl^–1^) and used to assess cell volume from measured capacitance in subsequent experiments.

**Table 1:** Key changes in Ca^2+^ handling in the old atria

	Young	Old	% Change during ageing	*P* value	% Change by nicardipine
Cell length ( μm)	127 ± 1.6	136 ± 2.4	↑7 ± 2%	*P *< 0.01, *n *= 223–343 cells 17–23 animals	–
Cell width ( μm)	15.1 ± 0.22	17.7 ± 0.4	↑17.2 ± 3%	*P *< 0.001, *n *= 223–343 cells 18–23 animals	–
Cell capacitance (pF)	62.7 ± 2.2	73.6 ± 3.5	↑17 ± 7%	*P *< 0.05, *n *= 41–53 cells 16–20 animals	–
Ca^2+^ transient amplitude (nmol l^−1^)	375 ± 33	280 ± 18	↓25 ± 8%	*P *< 0.05, *n *= 29–51 cells 15–23 animals	↓21 ± 3% *P *< 0.001
rate of decay of systolic Ca^2+^ (*k*_SYS_) (s^–1^)	8.5 ± 0.4	7.4 ± 0.3	↓13 ± 6%	*P *< 0.05, *n *= 29–51 cells 15–24 animals	–
Rate of caffeine transient decay (*k*_CAFF_) (s^–1^)	1.07 ± 0.10	1.08 ± 0.05	↔	NS, *n *= 22–38 cells 11–20 animals	–
*k*_SR_ (*k*_SYS_ – *k*_CAFF_) (s^–1^)	7.6 ± 0.5	6.2 ± 0.3	↓19 ± 7%	*P *< 0.05, *n *= 22–38 cells 11–20 animals	–
Buffer power	92.2 ± 7.6	135.5 ± 15	↑47 ± 20%	*P *< 0.05, *n *= 24–40 cells 14–21 animals	–
SR Ca content (μmol l^−1^)	75.3 ± 3.1	97.6 ± 4.2	↑29.6 ± 8%	*P *< 0.01, *n *= 43–52 cells 21–23 animals	↑22 ± 2% *P *< 0.01
Total Ca transient amplitude (μmol l^−1^)	32.9 ± 2.9	27.2 ± 2.3	↔	NS, *n *= 24–33 cells 14–19 animals	–
Peak *I*_Ca‐L_ (pA pF^–1^)	2.26 ± 0.11	1.83 ± 0.10	↓18.6 ± 7%	*P *< 0.05, *n *= 47–67 cells 19–25 animals	↓22.2 ± 2.5% *P *< 0.001
Integrated Ca^2+^ entry (μmol l^−1^)	0.76 ± 0.06	0.73 ± 0.05	↔	NS, *n *= 46–68 cells 18–25 animals	↓11 ± 4.8 *P *< 0.05
Fractional SR Ca^2+^ release	0.42 ± 0.04	0.29 ± 0.02	↓29.7 ± 9%	*P *< *0.01*, *n *= 24–33 cells 14–19 animals	↓25 ± 4.3%, *P *< 0.001

NS, not significant.

### Ageing decreases both the amplitude and rate of decay of the systolic Ca^2+^ transient

Typical Ca^2+^ transients recorded from single atrial myocytes under voltage clamp control are shown in Fig. 1 *C*. Ageing resulted in a 25 ± 8% decrease in Ca^2+^ transient amplitude (Fig. 1 *D* and Table 1), with no effect on diastolic Ca^2+^ (183 ± 11 *vs*. 166 ± 7.3 nmol l^−1^, not significant). Normalizing typical Ca^2+^ transients from experiments similar to Fig. 1 *C* reveals a decrease in the rate constant of decay of the systolic Ca^2+^ transient with age (Fig. 1 *F*). *k*_SYS_ was calculated by fitting a single exponential to the entire decay phase of the Ca^2+^ transient and was reduced by 13 ± 6% during ageing (*P *< 0.05) (Fig. 1 *G*).

The Ca^2+^ transient decays as Ca^2+^ is removed from the cytosol by SERCA (dominant pathway), NCX and minor pathways (e.g. plasma membrane Ca²⁺ ATPase and the mitochondrial Ca^2+^ uniporter). During the systolic Ca^2+^ transient, all Ca^2+^ removal pathways contribute; in contrast, during the caffeine‐evoked Ca^2+^ transient (Fig. 1 *E*), SERCA is negated as a result of the continued presence of caffeine. Thus, the canonical method to calculate the rate of Ca^2+^ removal by SERCA (*k*_SR_) is to fit the caffeine‐evoked Ca^2+^ transient with a single exponential and the rate constant of decay (*k*_CAFF_) is then subtracted from that of the systolic Ca^2+^ transient (*k*_SYS_). Normalizing caffeine‐evoked Ca^2+^ transients (Fig. 1 *H*) suggested that there was no effect of age on *k*_CAFF_, which is consistent with the data in Fig. 1 *I*. *k*_SR_, however, decreased by 19 ± 7% with age (Fig. 1 *I*). Subsequent experiments were designed to determine the mechanism responsible for decreases in (i) *k*_SR_ and (ii) Ca^2+^ transient amplitude.

### Slowed decay of systolic Ca^2+^ is not a result of the altered expression of key SR related proteins

**Figure 2:**
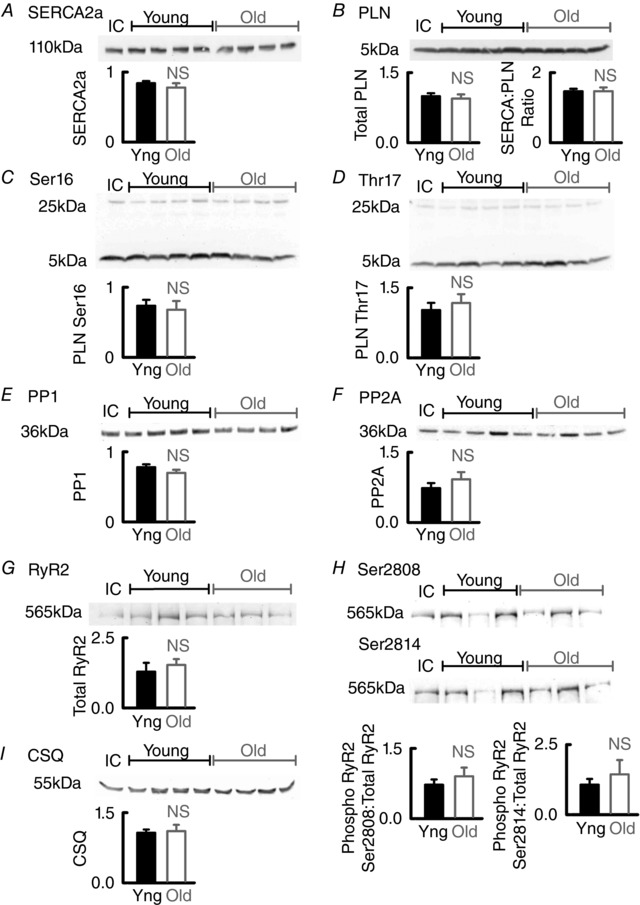
Expression levels and phosphorylation status of key SR related proteins are unaltered with age in the atria *A–D*, representative western blots for SERCA2a, total PLB and phosphorylated PLB (at Ser16 and Thr17 sites). *E* and *F*, representative western blots for PP1 and PP2A and *G* and *H*, RyR2 including phosphorylation at both Ser2808 and Ser2814 and calsequestrin (CSQ; *I*). Mean data are shown relative to the internal control for three repeats of 7–10 animals per group. NS, not significant.

Because decreased *k*_SR_ is often accounted for by decreased SERCA function, we first explored expression levels and phosphorylation status of relevant proteins. Surprisingly, protein levels of SERCA and PLB (including the SERCA:PLB ratio) remained unchanged with age (Fig. 2 *A* and *B*). Furthermore, we were unable to detect any change in PLB phosphorylation at Ser16 or Thr17, which is consistent with no change in the levels of the protein phosphatases PP1 and PP2A (Fig. 2 *C–F*). Taken together, these data suggest that SERCA function does not alter with age. An alternative explanation for the age‐associated decrease in *k*_SR_ would be an increase in leak of Ca^2+^ from the SR (Sankaranarayanan *et al*. 2016). However, protein levels of calsequestrin, RyR2 and the phosphorylation status of RyR at Ser 2808 and Ser2814 remained unchanged with age (Fig. 2 *G–I*). Thus, our data provide no evidence to support the hypothesis that either decreased SERCA function or increased SR leak can account for the decrease in the rate of decay of the Ca^2+^ transient. Because increased Ca buffering can slow the Ca^2+^ transient decay (Díaz *et al*. 2001b), we next investigated whether the age‐associated decrease in *k*_SR_ was buffering‐dependent.

### Increased intracellular Ca buffering underlies slowed decay of systolic Ca^2+^

**Figure 3:**
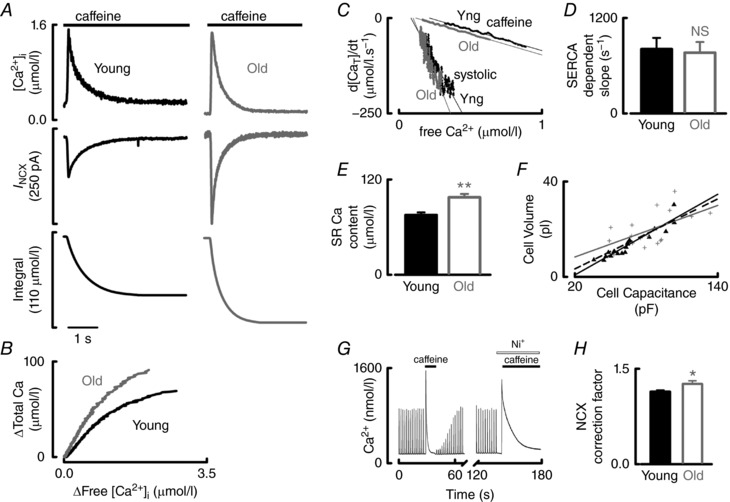
Both Ca buffering and SR Ca content are increased in old atrial myocytes *A*, quantitative assessment of free and total Ca in young (black) and old (grey) atrial myocytes. Caffeine‐evoked free Ca^2+^ transients (top) are associated with inward *I*_NCX_ (middle), which was integrated in a cumulative manner and corrected for cell volume and Ca^2+^ removal via pathways other than NCX to give a measure of total Ca (lower). *B*, typical cellular buffer curves showing the relationship between free and total Ca fit with a hyperbolic function (27–40 cells, 18–21 animals). *C*, typical relationships between the differential of the falling phase of total Ca plotted with respect to free Ca^2+^. Data are shown for both young and old systolic and caffeine‐evoked Ca^2+^ transients. Data points were fit with linear regressions. *D*, mean SERCA‐dependent slope calculated by subtracting the slope of the relationship for the caffeine‐evoked Ca^2+^ transient from that of the systolic transient as shown in (*C*) (*n *= 5–11 cells, 4–8 animals). *E*, mean SR Ca content as calculated from (*A*) (*n *= 43–52 cells, 23–24 animals). *F* – *H* describe the calculation of correction factors necessary to accurately calculate SR Ca content. *F*, cellular volume, calculated by confocal sections through a calcein loaded cell, plotted against the measured cellular capacitance for young (black triangles) and old (grey cross) cells. Young and old data sets were fit by linear regressions that were not significantly different for age. The dashed line therefore represents the fit to the whole data set. The mean correction factor was 4.80 ± 0.18 pF pl^–1^ (*n *= 17–19 cells, 5–8 animals). *G*, typical experimental time course for Ca^2+^ in an experiment designed to calculate the fraction of Ca^2+^ removed from the cytosol by factors other than SERCA and NCX. Single exponentials were fit to both the caffeine‐evoked Ca^2+^ transient and the caffeine transient in the presence of 10 mm Ni^+^ (to block NCX). The decay of the caffeine‐evoked Ca^2+^ transient in the presence of Ni^+^ was expressed as a fraction of that in the absence of Ni^+^ and a value of 1 added to generate correction factors; 1.14 ± 0.03 and 1.26 ± 0.05 for young and old atrial myocytes, respectively, shown in (*H*) (*n *= 20 cells, 8–10 animals). NS, not significant. ^*^ *P *< 0.05, ^**^ *P *< 0.01.

Experiments such as that shown in Fig. 3 *A* were used to calculate intracellular Ca buffering. Application of caffeine results in Ca^2+^ release from the SR into the cytosol where the Ca^2+^ indicator (Fluo‐5F) records the change in free Ca^2+^ (Fig. 3 *A*, upper). SERCA is effectively disabled in the continued presence of caffeine and Ca^2+^ is removed from the cell largely by NCX (Fig. 3 *A*, middle). Because, upon caffeine application, a small amount of Ca^2+^ is removed by pathways other than NCX, this must be accounted for to accurately calculate the change of (Δ) total Ca. Correction factors were determined in a separate series of experiments for young and old atrial myocytes (see Methods) (Fig. 3 *G* and *H*). The corrected integral of *I*_NCX_ thus gives a measure of the change in total cellular Ca (free Ca^2+^ plus bound Ca) (Fig. 3 *A*, lower).

Total Ca was plotted against free Ca^2+^ over the falling phase of the caffeine‐evoked Ca^2+^ transient (Trafford *et al*. 1999) to produce buffer curves (Fig. 3 *B*) and the data fitted with a hyperbolic function (eqn 2). The affinity for Ca^2+^ of the buffers increased with age; *K*_d_ decreases in the old atria (2.78 ± 0.4 *vs*. 1.73 ± 0.2 μmol l^−1^, *P *< 0.01) with no change in the maximum buffering capacity (*B*_max_, 336 ± 49 *vs*. 287 ± 30 μmol l^−1^, not significant, *n *= 27–40 cells from 18–21 animals). The buffering power (eqn 3) also increased with age (Table 1) (*P *< 0.05), suggesting that increased Ca buffering contributes to the slowed decay of the Ca^2+^ transient.

Although the protein data shown in Fig. 2 suggest that SERCA function is unchanged with age, we have tested this functionally. To remove the effects of buffering and to establish the contribution of SERCA alone, free Ca^2+^ was converted to total Ca using eqn (2). *B*_max_ and *K*_d_ values from the cells' own Ca buffer curve calculations were used. The falling phase of total Ca for both the systolic Ca^2+^ and caffeine‐evoked Ca^2+^ transients were differentiated with respect to time (d[Ca_T_]/dt) to give a rate of Ca^2+^ removal *independent* of Ca buffering and then plotted against free Ca^2+^ (Fig. 3 *C*). Data in Fig. 3 *C* (where the effects of Ca buffering were removed) were fit with linear regressions and the gradient of fits to the caffeine‐evoked Ca^2+^ transient subtracted from that of the systolic Ca^2+^ transient. The resultant value describes the SERCA‐dependent slope (independent of Ca buffering) and this was unchanged with age (Fig. 3 *D*). Thus, decreased *k*_SR_ is not the result of a decrease in SERCA function but rather increased Ca buffering in the old atria slows the decay of the Ca^2+^ transient. The mechanism underlying the decrease in Ca^2+^ transient amplitude was investigated next.

### SR Ca content increases with age

The corrected integral of *I*_NCX_ (Fig. 3 *A*, lower) gives a measure of the amount of Ca^2+^ that was stored within the SR. Thus, we aimed to determine whether decreased SR load underlies the decrease in Ca^2+^ transient amplitude with age. We found, however, that SR Ca content was actually increased with age by 29.6 ± 8% (75.3 ± 3.1 to 97.6 ± 4.2 μmol l^−1^, *P *< 0.001, *n *= 43–52 cells from 23–24 animals) (Fig. 3 *E* and Table 1). Thus, the observed age‐associated increase in SR Ca content cannot explain the decrease in Ca^2+^ transient amplitude. Because increased Ca buffering explains decreased systolic decay, we next aimed to determine whether it could also explain the decrease in Ca^2+^ transient amplitude in old atria.

### Increased Ca buffering underlies decreased Ca^2+^ transient amplitude in the old atria

**Figure 4:**
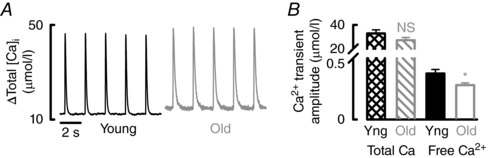
Total Ca transient amplitude is unaltered with age *A*, representative systolic Ca^2+^ transients converted to total Ca in young (black) and old (grey) atrial myocytes. *B*, mean data for total Ca transient amplitude and free Ca^2+^ transient amplitude (*n *= 24–33 cells, 14–19 animals). NS, not significant. ^*^ *P *< 0.05

Increased intracellular Ca buffering results in a smaller rise of free Ca^2+^ for a given addition of total Ca in old *vs*. young atrial myocytes and therefore decreases Ca^2+^ transient amplitude in older atria. We estimated the contribution of increased Ca buffering to the reduction in Ca^2+^ transient amplitude by converting free Ca^2+^ to total Ca using the calculated buffering parameters of cells. In contrast to the decrease in amplitude of free Ca^2+^, the amplitude of the total Ca transient did not alter with age (Fig. 4 *A* and *B* and Table 1). This data suggest that increased Ca buffering is responsible for decreased Ca^2+^ transient amplitude and the slowed decay of systolic Ca^2+^ in the old atria.

**Figure 5:**
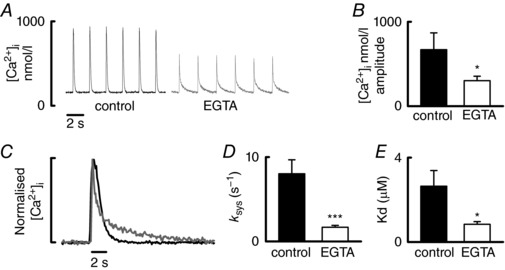
Increasing Ca buffering with EGTA decreases the amplitude and rate of decay of the systolic Ca^2+^ transient in young atrial cells *A*, typical Ca^2+^ transients recorded from control cells (left) and cells loaded with EGTA‐AM (right). *B*, mean data for Ca^2+^ transient amplitude. *C*, normalized Ca^2+^ transients from (*A*) to show the fast and slow components of decay in the presence of EGTA. The slow component represents Ca^2+^ unbinding from buffers. *D*, mean data for the rate of decay of the slow component. *E*, mean data for *K*_d_ confirming increased Ca^2+^ buffering in the presence of EGTA.

To confirm whether increased Ca buffering mimics the effects of ageing on the systolic Ca^2+^ transient, we artificially increased buffering in young atrial myocytes with EGTA in a separate series of experiments (Fig. 5 *A*). EGTA decreased both the amplitude of the systolic Ca^2+^ transient (Fig. 5 *B*) and the rate of decay of systolic Ca^2+^, as shown by the normalized records (Fig. 5 *C*) and mean data in Fig. 5 *D*. It is important to note that, as shown previously, increasing Ca buffering with EGTA results in fast and slow Ca^2+^ decay kinetics (Díaz *et al*. 2001a). The fast component of decay represents Ca^2+^ binding to EGTA, whereas the slow component represents the rate that Ca^2+^ is taken back from all buffers to be pumped back into the SR and out of the cell (Díaz *et al*. 2001a). We have therefore considered the slow component only in Fig. 5 *C* and *D*. An increase in Ca buffering was confirmed by a decrease in *K*_d_ (Fig. 5 *E*) which presumably resulted from the addition of a buffer of higher affinity than the cells endogenous buffers. Our data suggest that increasing Ca buffering recapitulates the effects of ageing on the systolic Ca^2+^ transient.

However, increased buffering cannot explain (i) the increase in SR Ca load or (ii) the lack of change in the amplitude of the total Ca transient despite an increase in SR Ca load. Because changes in *I*_Ca‐L_ can alter SR function, we next set out to determine any role for *I*_Ca‐L_ in age‐associated atrial remodelling.

### *I*_Ca‐L_ is decreased in the old atria with no change in integrated Ca^2+^ entry

**Figure 6:**
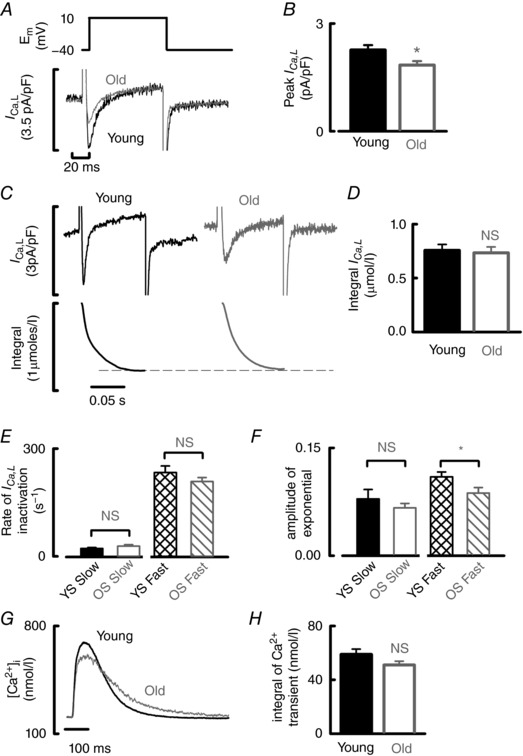
Peak *I*_Ca‐L_ is decreased but integrated Ca^2+^ entry maintained in old atrial myocytes *A*, voltage step (upper) used to elicit *I*_Ca‐L_ (lower) in young (black) and old (grey) atrial myocytes_._ *B*, mean data for peak *I*_Ca‐L_ (*n *= 47–67 cells, 19–25 animals). *C*, typical *I*_Ca‐L_ from young and old atrial myocytes (upper) and integrated *I*_Ca‐L_ over time (lower). Dotted line highlights the unchanged *I*_Ca‐L_ integral with age as illustrated in the mean data shown in (*D*). *E*, mean data for the rate of inactivation of *I*_Ca‐L_ fit using a double exponential with a fast and slow component presumably representing Ca^2+^ and voltage‐dependent inactivation (32–57 cells, 14–27 animals). *F*, amplitude of the double exponential during *I*_Ca‐L_ inactivation for both fast and slow components (32–57 cells, 14–27 animals). *G*, overlaid systolic Ca^2+^ transients from young (black) and old (grey) atrial myocytes. *H*, mean integral of the systolic Ca^2+^ transient (nmol l^–1^) (29–51 cells, 15–23 animals). NS, not significant. ^*^ *P *< 0.05.

Peak *I*_Ca‐L_ was reduced by 18.6 ± 7% in old atrial myocytes (Fig. 6 *A* and *B*). Integrated Ca^2+^ entry, however, was unaltered (Fig. 6 *C* and *D*) suggesting slowed *I*_Ca‐L_ inactivation in the old atria. It was not possible to fit *I*_Ca‐L_ decay with a single exponential, making any comparison between young and old data sets complicated. Slowed *I*_Ca‐L_ inactivation was not resolved by calculating the absolute rate of inactivation of exponents in Fig. 6 *E*. However, the decreased amplitude of the fast component of *I*_Ca‐L_ inactivation with age (Fig. 6 *F*) is consistent with slower *I*_Ca‐L_ inactivation. Example traces in Fig. 6 *C* were chosen to highlight slowed kinetics of the fast component of decay in the old cell, which, together with the decrease in peak, maintains the current integral.

Previously, we have shown that decreasing atrial *I*_Ca‐L_ results in an increase in SR Ca content (Clarke *et al*. 2015). In the present study, we have repeated that same experiment to determine whether the level by which *I*_Ca‐L_ is reduced during ageing (∼19% during ageing compared to ∼36% in heart failure) is sufficient to explain the increase in SR Ca content with age. Here, the age‐associated decrease in peak *I*_Ca‐L_ was mimicked in young atrial cells by pharmacological inhibition of *I*_Ca‐L_ with nicardipine to the levels observed in old animals (*I*_Ca,L_ was reduced by 22.2 ± 2.5%; 2.03 ± 0.21 to 1.60 ± 0.2 pA pF^–1^, *n *= 10 cells from four animals; data not shown). SR Ca content was assessed from the integral of the caffeine‐evoked NCX current in young atrial cells in the absence and presence of nicardipine. Reducing *I*_Ca‐L_ with nicardipine increased SR Ca content by 22 ± 2% (68.4 ± 5.3 to 82.6 ± 6.1 μmol l^−1^, *n *= 8 cells; data not shown) compared to 29.6 ± 8% during ageing. The discrepancy between these values is probably a result of the nicardipine‐induced decrease in integrated Ca^2+^ entry (0.40 ± 0.09 to 0.34 ± 0.07 μmol l^−1^, *P *< 0.05, *n *= 10 cells from four animals; data not shown), which was maintained during ageing. Thus, we suggest that the age‐associated increase in SR Ca load is explained by decreased peak and maintained integral of *I*_Ca‐L_.

Accordingly, we investigated whether decreased peak *I*_Ca‐L_ could explain why the total Ca transient amplitude was maintained during ageing despite an increase in SR Ca load. First, we calculated fractional SR Ca release using (Δ Ca total – integrated *I*_Ca‐L_)/SR Ca content (eqn 5). Fractional release decreased with age (Table 1); we next investigated whether this was a result of decreased peak *I*_Ca‐L_ rather than any change in the gain of excitation–contraction coupling (eqn 4). Gain, or the amount of Ca^2+^ released from the SR per unit *I*_Ca‐L_ trigger, was not altered by age (14.5 ± 1.4 *vs*. 15.3 ± 1.4, not significant, *n *= 24–33 cells from 14–19 animals) when assessed using (Δ Ca total – integrated *I*_Ca‐L_)/peak *I*_Ca‐L_ despite increased SR Ca load. Thus, our data suggest that SR Ca release is maintained during ageing because decreased fractional release opposes increased SR Ca load, maintaining the total Ca transient.

**Figure 7:**
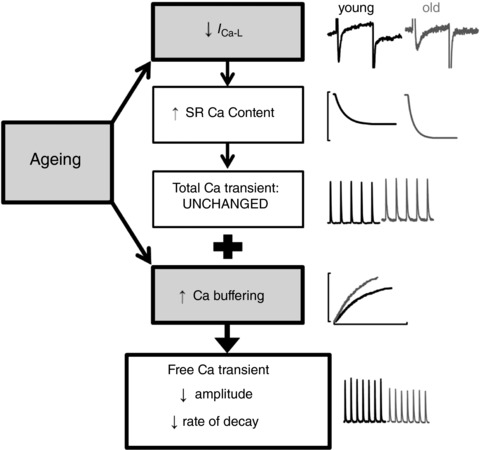
Key mechanisms underlying altered Ca^2+^ transient properties Key mechanistic links defining age‐associated remodelling of the systolic Ca^2+^ transient in the sheep atria. In summary, ageing increases Ca buffering and decreases peak *I*_Ca‐L_. Remodelling of *I*_Ca‐L_ increases SR Ca content but decreases fractional SR Ca^2+^ release. The net effect of decreased fractional release with increased SR load is maintained SR Ca release and thus unaltered total Ca transient amplitude. Increased Ca buffering results in slower and smaller Ca^2+^ transients in old atrial myocytes.

Finally, because integrated Ca^2+^ entry via *I*_Ca‐L_ does not change with age, Ca^2+^ efflux must also be maintained to preserve flux balance. Although a decrease in the amplitude of the free Ca^2+^ transient would be expected to decrease Ca^2+^ efflux, a slowing of decay would be expected to increase efflux. To assess the net effect, the integrated area underneath the free Ca^2+^ transient was calculated and found not to change with age (Fig. 6 *G* and *H*). Thus, during ageing, the effects of increased buffering (decreased amplitude and slowed decay) act in concert to maintain Ca^2+^ efflux. A summary of the age‐associated changes influencing systolic Ca^2+^ is provided in Fig. 7.

## Discussion

In the present study, we have described and elucidated mechanisms for alterations in atrial intracellular Ca^2+^ handling in old sheep. We show that ageing resulted in: (i) cellular hypertrophy; (ii) decreased amplitude and rate of decay of the systolic Ca^2+^ transient brought about by an increase in intracellular Ca buffering; (iii) decreased peak *I*_Ca‐L_ with maintained integrated Ca^2+^ entry; and (iv) increased SR Ca load arising as a consequence of decreased peak *I*_Ca‐L_.

### Ageing leads to hypertrophy of atrial myocytes

Healthy ageing in sheep results in an increase in atrial cell dimensions and cellular capacitance. Increasing cellular capacitance with age has been reported previously in the dog right atrium (Dun *et al*. 2003) and left atrial enlargement has been observed in elderly healthy humans (Boyd *et al*. 2011). A recent human study, however, reported unchanged atrial and cellular dimensions during ageing (Herraiz *et al*. 2015), which may reflect confounding factors from underlying disease. Interestingly, increasing left atrial size is one of the strongest risk factors for AF (Vaziri *et al*. 1994; Tsang *et al*. 2006). Although we have not measured atrial size in the present study, increased diastolic blood pressure and ventricular collagen content previously reported in our sheep model of ageing (Horn *et al*. 2012) may promote left atrial hypertrophy. Interestingly increased left atrial size is correlated with pericardial adipose tissue (Greif *et al*. 2013) and both atrial size and peri‐atrial fat are potential risk factors for AF (Tsang *et al*. 2006; Haemers *et al*. 2017).

### Increased Ca buffering underpins decreased amplitude and slowed decay of systolic Ca^2+^

The amount of Ca^2+^ stored in the SR and the function of SERCA are, at least in the ventricle, the primary determinants of Ca^2+^ transient amplitude and rate of systolic decay, respectively. Surprisingly, in the present study, the decrease in Ca^2+^ transient amplitude with age was associated with an increase in SR Ca content and slowed decay associated with no change in SERCA function or expression. Thus, our data suggest that factors other than SR function play a role in remodelling systolic Ca^2+^ in the aged atria.

Less than 1% of Ca^2+^ in the cytosol is free; the rest is bound to intracellular buffers (Berlin *et al*. 1994; Trafford *et al*. 1999) e.g. the myofilaments and SERCA (Briston *et al*. 2014); a review is provided by Bers (2001). In the present study, we find that intracellular Ca buffering is increased in old atria. This, rather than any change in the underlying total Ca, is responsible for the decrease in amplitude of the free Ca^2+^ transient. As well as decreasing Ca^2+^ transient amplitude, increased buffering slows the decay of systolic Ca^2+^ both in the atria (as shown in the present study by increasing Ca buffering with EGTA) and in the ventricle, as reported previously (Díaz *et al*. 2001a, b). The role of buffering in age‐associated remodelling is substantiated because increasing Ca buffering in young atrial myocytes recapitulates the phenotype of the old atrial Ca^2+^ transient (i.e. decreased amplitude and rate of decay of systolic Ca^2+^).

In the steady‐state, the Ca^2+^ efflux from the cell must equal Ca^2+^ influx. The integrated Ca^2+^ influx is unaffected by ageing (Fig. 6) and therefore the efflux must be constant. Previously, we have shown that efflux can be increased by increasing diastolic Ca^2+^ (Dibb *et al*. 2007); however, diastolic Ca^2+^ did not alter with age. Thus, this constancy arises because, although increased buffering decreases the amplitude of the free Ca^2+^ transient, which would decrease efflux, it also slows the rate constant of decay, thereby promoting efflux. Integrating the Ca^2+^ transient takes both of these factors into consideration. The integral is unaffected by age and, if we assume that Ca^2+^ efflux is proportional to [Ca^2+^]_i_, then the constancy of the integral would result in unchanged Ca^2+^ efflux with ageing.

We have previously shown that Ca buffering in the rat atrium is ∼3‐fold higher than in the ventricle as a result of elevated SERCA expression and function and thus increased affinity for Ca^2+^ (Walden *et al*. 2009). Interestingly, rapid atrial pacing has also been shown to increase atrial Ca buffering, with important consequences for intracellular Ca^2+^ signalling (Greiser *et al*. 2014). Despite its importance, we are unaware of any studies measuring Ca buffering during ageing of the atria or ventricle and thus this represents a novel mechanism of age‐associated atrial remodelling. Taken together, these data suggest that Ca buffering is an important regulator of intracellular Ca^2+^ in the atria.

It is unclear what is responsible for the increase in buffering with age. We report no change in *B*_max_ but a decrease in the *K*_d_ of Ca^2+^ binding, suggesting that the total amount of buffers is unchanged, whereas the affinity of the buffers for Ca^2+^ is increased. The two major Ca^2+^ buffers are troponin and SERCA. We found that SERCA expression and function are unaffected. It is therefore possible that an increase in the affinity of troponin for Ca^2+^ occurs. An increase in the affinity of troponin for Ca^2+^ would help to activate myofilaments at lower Ca^2+^ levels and hence maintain contraction in the aged atria (Knollmann *et al*. 2003). However, we find *in vivo* that the left atrial fractional area change is decreased with ageing (M. A. Horn, personal communication), suggesting that any increase in the affinity of troponin for Ca^2+^ is not sufficient to maintain atrial contraction during ageing.

### The role of decreased *I*_Ca‐L_ in the aged atria

Initially, decreased peak *I*_Ca‐L_ might appear to be an obvious candidate accounting for the decrease of Ca^2+^ transient amplitude with age. However, during ageing, the decrease of peak Ca^2+^ current is not accompanied by any change of integrated influx. The fact that the integrated influx is constant means that, in the steady‐state, the efflux must also be unchanged. This requires (irrespective of what happens to the peak current) that the Ca^2+^ transient, or the amount of Ca^2+^ seen by NCX, must be unaltered. In older atria, efflux is maintained by slowed decay of systolic Ca^2+^ as discussed above.

The decrease in peak *I*_Ca‐L_ does, however, result in an increase in SR Ca content in old atrial cells. Peak *I*_Ca‐L_ acts as the trigger for SR Ca release, whereas the integral acts to load the cell, and thus the SR, with Ca^2+^. Decreased peak *I*_Ca‐L_ decreases the fractional SR Ca release, resulting in less Ca^2+^ being available for extrusion from the cell, which presumably acts to increase SR Ca content. Because the integral of *I*_Ca‐L_ is unaltered by age, this effect is unopposed in the old atria.

Decreased peak *I*_Ca‐L_ increases SR Ca content to a level where the combination of decreased peak *I*_Ca‐L_ and increased SR Ca content results in the same release of Ca^2+^ from the SR in older as in younger atria (Fig. 7). Thus, the amplitude of the total Ca transient (i.e. where the effects of buffering have been removed) is unchanged and we conclude that the observed changes of *I*_Ca‐L_ cannot account for the decreased free Ca^2+^ transient amplitude with age. Finally, the constancy of integrated Ca^2+^ entry on the L‐type Ca^2+^ current probably arises because decreased systolic Ca^2+^ decreases the rate of Ca^2+^‐dependent inactivation of *I*_Ca‐L_ (Trafford *et al*. 1997).

The observed decrease of *I*_Ca‐L_ is consistent with previous work performed in the atria of older dogs (Dun *et al*. 2003; Gan *et al*. 2013). In the right atrium, decreased *I*_Ca‐L_ was only apparent when Ca^2+^‐dependent inactivation was inhibited (Dun *et al*. 2003). The few studies investigating *I*_Ca‐L_ in humans show disparate results. Some show no age‐dependant change in peak *I*_Ca‐L_ (Roca *et al*. 1996; Tipparaju *et al*. 2004), whereas a more recent study reports that *I*_Ca‐L_ decreases with age (Herraiz *et al*. 2015). The disparity in human data may relate to comorbidities, drug treatment or the absolute ages of the young and old cohorts. All of these factors can affect atrial t‐tubule density (Dibb *et al*. 2009; Lenaerts *et al*. 2009) and systolic Ca^2+^, highlighting the importance of healthy ageing studies. Interestingly, however, we have found that ageing increases ventricular peak *I*_Ca‐L_ and, consequently, the amplitude of the systolic Ca^2+^ transient (Dibb *et al*. 2004), suggesting that age‐associated remodelling of Ca^2+^ handling is chamber‐specific.

### The significance of an age‐associated increase in SR Ca content

Ageing increases SR Ca content in the sheep atria as a result of a decrease in peak *I*_Ca‐L_. This is similar to other pro‐arrhythmic states such as heart failure in which increased atrial SR Ca content has also been reported (Yeh *et al*. 2008; Clarke *et al*. 2015). These findings, however, contrast with one study reporting a decrease in SR Ca content in old human atria (Herraiz *et al*. 2015). One explanation for the disparity could be that human samples came from patients with disease undergoing cardiac surgery, whereas, in the present study, all sheep were free from detectable cardiovascular disease. Second, the youngest cohort in the human study was <55 years of age and may not be comparable with young sheep, which were considered to be equivalent to young adults. Finally, in the human data above, the male sex predominates, whereas the present study focuses on female sheep. Interestingly, an age‐associated increase in SR Ca content has been observed in the ventricle of female but not male rats, whereas *I*_Ca‐L_ decreased in both sexes (Howlett, 2010). The increased SR load in the atria of old sheep could therefore be sex‐dependent. The lack of change in SERCA function, expression or phosphorylation status of SERCA and PLB, or the ratio between the two, is consistent with increased Ca buffering defining slowed Ca^2+^ transient decay in old atria. However, other studies have found atrial SERCA function or expression to decrease with age (Cain *et al*. 1998; Schmidt *et al*. 2000; Herraiz *et al*. 2015). While SERCA activity is also decreased in the old rodent ventricle, a number of studies show unaltered SERCA or PLB expression (Feridooni *et al*. 2015).

It is important to emphasize that, had we not considered Ca buffering in the present study, we would also have calculated a decrease in SERCA function. We found the rate of decay of systolic Ca^2+^ (*k*_SYS_) was decreased in the old atria, as was the rate constant following correction for Ca^2+^ removal by pathways other than SERCA (*k*_SR_). Initially, this might suggest an age‐associated decrease in SERCA function. However, when SERCA activity was calculated directly from changes of total Ca, SERCA was actually unaltered.

### The relevance of abnormal calcium handling to age‐associated atrial fibrillation

Although we have not investigated the propensity for arrhythmias in the present study, the abnormalities in Ca^2+^ handling that we report in myocytes from old sheep could promote AF by several mechanisms. First, the increase in SR Ca content could increase the propensity to spontaneous diastolic Ca^2+^ release, leading to delayed after depolarisations (Venetucci *et al*. 2007) as observed in AF (Voigt *et al*. 2012; Voigt *et al*. 2014). Furthermore, the combination of increased SR Ca content with decreased *I*_Ca‐L_ leads to AP alternans in rat ventricular myocytes (Li *et al*. 2009). AP alternans, in turn, can promote AF by causing a dispersion of repolarisation favouring wavebreak (Narayan *et al*. 2011). However, in atrial myocytes, decreased *I*_Ca‐L_ may protect against alternans but an elevated SR load result in irregular beat to beat patterns of intracellular Ca^2+^ that are suggested to precede arrhythmias (Llach *et al*. 2011). Disordered Ca buffering itself has been associated with a heightened susceptibility to arrhythmias, although the mechanisms underlying this remain unclear (Knollmann *et al*. 2003). Finally, although a decrease in *I*_Ca‐L_ and Ca^2+^ transient amplitude might be expected to shorten action potential duration and thereby promote arrhythmias by decreasing the atrial effective refractory period, ageing is however associated with longer atrial AP durations in dogs (Anyukhovsky *et al*. 2005).

In addition to Ca^2+^ handling, the balance of autonomic tone changes with age. Sympathetic nerve activity increases with age in man accompanied by a decrease in heart rate variability (Abhishekh *et al*. 2013). Decreased heart rate variability in old sheep (Horn *et al*. 2016) suggests that an age‐associated increase in sympathetic tone is preserved across species. Sympathetic tone, activating β‐adrenoceptors, probably exacerbates the age‐associated increase in SR Ca content (Briston *et al*. 2011) without affecting Ca buffering (Briston *et al*. 2014) and may therefore increase the probability of spontaneous SR Ca release (Venetucci *et al*. 2007).

Similarly, it is relevant that a decrease in *I*_Ca‐L_ increases SR Ca content in the sheep atria at the point of heart failure (Clarke *et al*. 2015). Because heart failure and AF are primarily diseases of the elderly, a pathological reduction in *I*_Ca,L_ in addition to an age‐associated *I*_Ca,L_ reduction could exacerbate any increase in SR Ca content and thus the probability of spontaneous SR Ca release and AF (Voigt *et al*. 2012; Voigt *et al*. 2014).

### Limitations

Data were collected using the perforated patch technique to mimic the physiological situation as closely as possible. Therefore, intracellular Ca^2+^ was measured using Fluo‐5F AM. The possibility that loading with this fluorophore was uneven between age groups was minimized by taking great care to standardize loading conditions and using probenicid to prevent dye leakage. In the present study, the amplitude of the *F*_max_ was unaltered with age and showed no relationship with buffer power. Therefore, our data support equal loading of Fluo‐5F between age groups and no role for the fluorescence indicator in the age‐associated changes in Ca buffering. Direct loading of the fluorophore via the patch pipette was impractical because of the disadvantages of whole‐cell patch recording for the long experiments used in the present study, as well as the possibility that altering the intercellular milieu could itself alter Ca buffering.

Furthermore, in our experience, loading with the pentopotassium salt of Fluo‐5F (50 μm) slows the Ca^2+^ transient decay more than when loading with the AM ester, suggesting that, under our experimental conditions, the AM ester buffers Ca^2+^ less than the pentopotassium salt. We have estimated passive Ca^2+^ buffering of Fluo‐5F and we expect ∼9% of total Ca to be bound to the indicator in our experiments. We suggest this will have a negligible effect on our data and, because buffering by Fluo‐5F is unaltered by age, this will have no effect on our conclusions.

The experiments were performed using the perforated patch clamp technique and therefore incurred a higher access resistance than would have been the case if the experiments had been performed using the whole cell technique. Any effects of increased access resistance were minimized using the discontinuous (switch–clamp) voltage clamp technique. Given the differences in capacitance and known access resistance, even in the uncompensated state, any difference in voltage errors would be less than 1 mV in these experiments. In the present study, a holding potential of −40 mV was used to isolate *I*_Ca‐L_; however, this is not representative of the membrane potential during the action potential. We do not expect changes in membrane potential to alter Ca buffering, diastolic Ca^2+^ or the overall effect of ageing, although the absolute magnitude of *I*_Ca‐L_ and Ca^2+^ transient amplitude would be expected to be reduced in young and old cells at −40 *vs*. −80 mV (Dibb *et al*. 2007).

The work reported in the present study was collected from young and old female sheep. It is possible that the differences presented here could be sex‐ or species‐specific. However, a decrease in atrial *I*_Ca‐L_ has been reported in studies of dogs (Gan *et al*. 2013) and humans (Herraiz *et al*. 2015) of both sexes. This suggests that at least some aspects of Ca^2+^ handling are remodelled with age across species in both sexes.

### Conclusions

Our data highlights novel mechanisms by which atrial Ca^2+^ handling adapts with age, as summarized in Fig. 7. We suggest that increased intracellular Ca buffering decreases both the amplitude and rate of decay of the free Ca^2+^ transient in the old atria of female sheep. The age‐associated decrease in *I*_Ca‐L_ acts to increase SR Ca content but to decrease fractional release; thus, the amount of Ca^2+^ released from the SR and the amplitude of the total Ca transient are maintained.

## Additional information

### Competing interests

The authors declare that they have no competing interests.

### Author contributions

All authors approved the final version of the manuscript submitted for publication and agree to be accountable for all aspects of the work. KMD, DAE and AWT conceived the experiments and obtained funding. KMD, JLC, JDC and CMP conducted the experimental work.

### Funding

This work was supported by a British Heart Foundation Intermediate Research Fellowship (FS/09/002/26487; KMD), a University of Manchester Stepping Stone Award (KMD), British Heart Foundation Project Grants (PG/09/062 and PG/12/89/29970), a British Heart Foundation Clinical Research Training Fellowship (FS/12/34/29565; CMP), The BHF Michael Frazer PhD Studentship (FS/07/003) and a European Union 6th Framework STREP Project ‘Normacor’.

## References

[tjp12537-bib-0001] Abhishekh HA , Nisarga P , Kisan R , Meghana A , Chandran S , Trichur R & Sathyaprabha TN (2013). Influence of age and gender on autonomic regulation of heart. J Clin Monit Comput 27, 259–264. 23297094 10.1007/s10877-012-9424-3

[tjp12537-bib-0002] Anyukhovsky EP , Sosunov EA , Chandra P , Rosen TS , Boyden PA , Danilo P, Jr. & Rosen MR (2005). Age‐associated changes in electrophysiologic remodeling: a potential contributor to initiation of atrial fibrillation. Cardiovasc Res 66, 353–363. 15820204 10.1016/j.cardiores.2004.10.033

[tjp12537-bib-0003] Berlin JR , Bassani JWM & Bers DM (1994). Intrinsic cytosolic calcium buffering properties of single rat cardiac myocytes. Biophys J 67, 1775–1787. 7819510 10.1016/S0006-3495(94)80652-6 PMC1225540

[tjp12537-bib-0004] Bers DM (2001). Excitation‐Contraction Coupling and Cardiac Contractile Force, (2 ed.) Kluwer Academic Publishers, Dordrecht.

[tjp12537-bib-0005] Boyd AC , Schiller NB , Leung D , Ross DL & Thomas L (2011). Atrial dilation and altered function are mediated by age and diastolic function but not before the eighth decade. JACC Cardiovasc Imaging 4, 234–242. 21414570 10.1016/j.jcmg.2010.11.018

[tjp12537-bib-0006] Briston SJ , Caldwell JL , Horn MA , Clarke JD , Richards MA , Greensmith DJ , Graham HK , Hall MC , Eisner DA , Dibb KM & Trafford AW (2011). Impaired β‐adrenergic responsiveness accentuates dysfunctional excitation contraction coupling in an ovine model of tachypacing induced heart failure. J Physiol (Lond) 589, 1367–1382. 21242250 10.1113/jphysiol.2010.203984 PMC3082097

[tjp12537-bib-0007] Briston SJ , Dibb KM , Solaro RJ , Eisner DA & Trafford AW (2014). Balanced changes in Ca buffering by SERCA and troponin contribute to Ca handling during beta‐adrenergic stimulation in cardiac myocytes. Cardiovasc Res 104, 347–354. 25183792 10.1093/cvr/cvu201 PMC4240166

[tjp12537-bib-0008] Cain BS , Meldrum DR , Joo KS , Wang JF , Meng X , Cleveland JC Jr , Banerjee A & Harken AH (1998). Human SERCA2a levels correlate inversely with age in senescent human myocardium. J Am Coll Cardiol 32, 458–467. 9708476 10.1016/s0735-1097(98)00233-2

[tjp12537-bib-0009] Caldwell JL , Smith CE , Taylor RF , Kitmitto A , Eisner DA , Dibb KM & Trafford AW (2014). Dependence of cardiac transverse tubules on the BAR domain protein amphiphysin II (BIN‐1). Circ Res 115, 986–996. 25332206 10.1161/CIRCRESAHA.116.303448 PMC4274343

[tjp12537-bib-0010] Cheng H , Lederer WJ & Cannell MB (1993). Calcium sparks: elementary events underlying excitation‐contraction coupling in heart muscle. Science 262, 740–744. 8235594 10.1126/science.8235594

[tjp12537-bib-0011] Clarke JD , Caldwell JL , Horn MA , Bode EF , Richards MA , Hall MC , Graham HK , Briston SJ , Greensmith DJ , Eisner DA , Dibb KM & Trafford AW (2015). Perturbed atrial calcium handling in an ovine model of heart failure: potential roles for reductions in the L‐type calcium current. J Mol Cell Cardiol 79C, 169–179. 10.1016/j.yjmcc.2014.11.017 PMC4312356 25463272

[tjp12537-bib-0012] Di BL , Burkhardt JD , Mohanty P , Sanchez J , Mohanty S , Horton R , Gallinghouse GJ , Bailey SM , Zagrodzky JD , Santangeli P , Hao S , Hongo R , Beheiry S , Themistoclakis S , Bonso A , Rossillo A , Corrado A , Raviele A , Al‐Ahmad A , Wang P , Cummings JE , Schweikert RA , Pelargonio G , Dello RA , Casella M , Santarelli P , Lewis WR & Natale A (2010). Left atrial appendage: an underrecognized trigger site of atrial fibrillation. Circulation 122, 109–118. 20606120 10.1161/CIRCULATIONAHA.109.928903

[tjp12537-bib-0013] Díaz ME , Graham HK & Trafford AW (2004). Enhanced sarcolemmal Ca^2+^ efflux reduces sarcoplasmic reticulum Ca^2+^ content and systolic Ca^2+^ in cardiac hypertrophy. Cardiovasc Res 62, 538–547. 15158146 10.1016/j.cardiores.2004.01.038

[tjp12537-bib-0014] Díaz ME , Trafford AW & Eisner DA (2001a). The effects of exogenous Ca^2+^ buffers on the systolic Ca^2+^ transient in rat ventricular myocytes. Biophys J 80, 1915–1925. 11259304 10.1016/S0006-3495(01)76161-9 PMC1301380

[tjp12537-bib-0015] Díaz ME , Trafford AW & Eisner DA (2001b). The role of intracellular Ca^2+^ buffers in determining the shape of the systolic Ca^2+^ transient in cardiac ventricular myocytes. Pflügers Archiv 442, 96–100. 11374074 10.1007/s004240000509

[tjp12537-bib-0016] Dibb KM , Clarke JD , Horn MA , Richards MA , Graham HK , Eisner DA & Trafford AW (2009). Characterization of an extensive transverse tubular network in sheep atrial myocytes and its depletion in heart failure. Circ Heart Fail 2, 482–489. 19808379 10.1161/CIRCHEARTFAILURE.109.852228

[tjp12537-bib-0017] Dibb KM , Eisner DA & Trafford AW (2007). Regulation of systolic [Ca^2+^]_i_ and cellular Ca^2+^ flux balance in rat ventricular myocytes by SR Ca^2+^, L‐type Ca^2+^ current and diastolic [Ca^2+^]_i_ . J Physiol (Lond) 585, 579–592. 17932152 10.1113/jphysiol.2007.141473 PMC2375487

[tjp12537-bib-0018] Dibb KM , Hagarty CL , Loudon ASI & Trafford AW (2005). Photoperiod dependent modulation of cardiac excitation contraction coupling in the Siberian hamster. Am J Physiol *Regul Integr Comp Physiol* 288, R607–R614. 10.1152/ajpregu.00612.2004 15528392

[tjp12537-bib-0019] Dibb KM , Rueckschloss U , Eisner DA , Isenberg G & Trafford AW (2004). Mechanisms underlying enhanced cardiac excitation contraction coupling observed in the senescent sheep myocardium. J Mol Cell Cardiol 37, 1171–1181. 15572047 10.1016/j.yjmcc.2004.09.005

[tjp12537-bib-0020] Dun W , Yagi T , Rosen MR & Boyden PA (2003). Calcium and potassium currents in cells from adult and aged canine right atria. Cardiovasc Res 58, 526–534. 12798425 10.1016/s0008-6363(03)00288-8

[tjp12537-bib-0021] Ennos R (2000). Choosing tests and designing experiments In Statistical and Data Handling Skills in Biology, pp. 84–99. Pearson Education Ltd, Harlow.

[tjp12537-bib-0022] Feridooni HA , Dibb KM & Howlett SE (2015). How cardiomyocyte excitation, calcium release and contraction become altered with age. J Mol Cell Cardiol 83, 62–72. 25498213 10.1016/j.yjmcc.2014.12.004

[tjp12537-bib-0023] Gan TY , Qiao W , Xu GJ , Zhou XH , Tang BP , Song JG , Li YD , Zhang J , Li FP , Mao T & Jiang T (2013). Aging‐associated changes in L‐type calcium channels in the left atria of dogs. Exp Ther Med 6, 919–924. 24137290 10.3892/etm.2013.1266 PMC3797308

[tjp12537-bib-0024] Gelman A & Hill J (2007). Data Analysis Using Regression and Mulitlevel/Hierachical Models Cambridge University Press, Cambridge.

[tjp12537-bib-0025] Graham HK & Trafford AW (2007). Spatial disruption and enhanced degradation of collagen with the transition from compensated ventricular hypertrophy to symptomatic congestive heart failure. Am J Physiol *Heart Circ Physiol* 292, H1364–H1372. 10.1152/ajpheart.00355.2006 17071734

[tjp12537-bib-0026] Greif M , von ZF , Wakili R , Tittus J , Becker C , Helbig S , Laubender RP , Schwarz W , D'Anastasi M , Schenzle J , Leber AW & Becker A (2013). Increased pericardial adipose tissue is correlated with atrial fibrillation and left atrial dilatation. Clin Res Cardiol 102, 555–562. 23584714 10.1007/s00392-013-0566-1

[tjp12537-bib-0027] Greiser M , Kerfant BG , Williams GS , Voigt N , Harks E , Dibb KM , Giese A , Meszaros J , Verheule S , Ravens U , Allessie MA , Gammie JS , van d, V , Lederer WJ , Dobrev D & Schotten U (2014). Tachycardia‐induced silencing of subcellular Ca^2+^ signaling in atrial myocytes. J Clin Invest 124, 4759–4772. 25329692 10.1172/JCI70102 PMC4347234

[tjp12537-bib-0028] Haemers P , Hamdi H , Guedj K , Suffee N , Farahmand P , Popovic N , Claus P , LePrince P , Nicoletti A , Jalife J , Wolke C , Lendeckel U , Jais P , Willems R & Hatem SN (2017). Atrial fibrillation is associated with the fibrotic remodelling of adipose tissue in the subepicardium of human and sheep atria. Eur Heart J 38, 53–61. 26612579 10.1093/eurheartj/ehv625

[tjp12537-bib-0029] Haissaguerre M , Jais P , Shah DC , Takahashi A , Hocini M , Quiniou G , Garrigue S , Le MA , Le MP & Clementy J (1998). Spontaneous initiation of atrial fibrillation by ectopic beats originating in the pulmonary veins. N Engl J Med 339, 659–666. 9725923 10.1056/NEJM199809033391003

[tjp12537-bib-0030] He J‐Q , Conklin MW , Foell JD , Wolff MR , Haworth RA , Coronado R & Kamp TJ (2001). Reduction in density of transverse tubules and L‐type Ca^2+^ channels in canine tachycardia‐induced heart failure. Cardiovasc Res 49, 298–307. 11164840 10.1016/s0008-6363(00)00256-x

[tjp12537-bib-0031] Herraiz A , Alvarez‐Garcia J , Llach A , Molina CE , Fernandes J , Ferrero A , Rodriguez C , Vallmitjana A , Benitez R , Padro J , Martinez‐Gonzalez J , Cinca J & Hove‐Madsen L (2015). Ageing is associated with deterioration of calcium homeostasis in isolated human right atrial myocytes. Cardiovasc Res 106, 76–86. 25712961 10.1093/cvr/cvv046 PMC4362404

[tjp12537-bib-0032] Horn MA , Bode EF , Borland SJ , Kirkwood GJ , Briston SJ , Richards MA , Dibb KM & Trafford AW (2016). Temporal development of autonomic dysfunction in heart failure: effects of age in an ovine rapid‐pacing model. J Gerontol A Biol Sci Med Sci 71, 1544–1552. 26707382 10.1093/gerona/glv217 PMC5106849

[tjp12537-bib-0033] Horn MA , Graham HK , Richards MA , Clarke JD , Greensmith DJ , Briston SJ , Hall MCS , Dibb KM & Trafford AW (2012). Age‐related divergent remodeling of the cardiac extracellular matrix in heart failure: collagen accumulation in the young and loss in the aged. J Mol Cell Cardiol 53, 82–90. 22516365 10.1016/j.yjmcc.2012.03.011

[tjp12537-bib-0034] Howlett SE (2010). Age‐associated changes in excitation‐contraction coupling are more prominent in ventricular myocytes from male rats than in myocytes from female rats. Am J Physiol Heart Circ Physiol 298, H659–H670. 19966062 10.1152/ajpheart.00214.2009

[tjp12537-bib-0035] Kirkwood TB (2008). A systematic look at an old problem. Nature 451, 644–647. 18256658 10.1038/451644a

[tjp12537-bib-0036] Knollmann BC , Kirchhof P , Sirenko SG , Degen H , Greene AE , Schober T , Mackow JC , Fabritz L , Potter JD & Morad M (2003). Familial hypertrophic cardiomyopathy‐linked mutant troponin T causes stress‐induced ventricular tachycardia and Ca^2+^‐dependent action potential remodeling. Circ Res 92, 428–436. 12600890 10.1161/01.RES.0000059562.91384.1A

[tjp12537-bib-0037] Lakatta EG (2015). So! What's aging? Is cardiovascular aging a disease? J Mol Cell Cardiol 83, 1–13. 25870157 10.1016/j.yjmcc.2015.04.005 PMC4532266

[tjp12537-bib-0038] Lenaerts I , Bito V , Heinzel FR , Driesen RB , Holemans P , D'hooge J , Heidbuchel H , Sipido KR & Willems R (2009). Ultrastructural and functional remodeling of the coupling between Ca^2+^ influx and sarcoplasmic reticulum Ca^2+^ release in right atrial myocytes from experimental persistent atrial fibrillation. Circ Res 105, 876–885. 19762679 10.1161/CIRCRESAHA.109.206276

[tjp12537-bib-0039] Li Y , Diaz ME , Eisner DA & O'Neill SC (2009). The effects of membrane potential, SR Ca content and RyR responsiveness on systolic Ca alternans in rat ventricular myocytes. J Physiol 587, 1283–1292. 19153161 10.1113/jphysiol.2008.164368 PMC2674997

[tjp12537-bib-0040] Llach A , Molina CE , Fernandes J , Padro J , Cinca J & Hove‐Madsen L (2011). Sarcoplasmic reticulum and L‐type Ca(2) channel activity regulate the beat‐to‐beat stability of calcium handling in human atrial myocytes. J Physiol 589, 3247–3262. 21521767 10.1113/jphysiol.2010.197715 PMC3145937

[tjp12537-bib-0041] Loughrey CM , MacEachern KE , Cooper J & Smith GL (2003). Measurement of the dissociation constant of Fluo‐3 for Ca^2+^ in isolated rabbit cardiomyocytes using Ca^2+^ wave characteristics. Cell Calcium 34, 1–9. 12767887 10.1016/s0143-4160(03)00012-5

[tjp12537-bib-0042] Moresco A , Haworth D , Orton C & Barrington G (2001). ECG of the Month. Atrial Fibrillation in a ram with abomasal emptying defect. J Am Vet Med Assoc 218, 1264–1266. 11330610

[tjp12537-bib-0043] Narayan SM , Franz MR , Clopton P , Pruvot EJ & Krummen DE (2011). Repolarization alternans reveals vulnerability to human atrial fibrillation. Circulation 123, 2922–2930. 21646498 10.1161/CIRCULATIONAHA.110.977827 PMC3135656

[tjp12537-bib-0044] Pope GW (1934). Determining the age of farm animals by their teeth In Department of Agriculture, Farmer's Bulletin, pp. 13 U.S. Dept. of Agriculture, Washington D.C.

[tjp12537-bib-0045] Richards MA , Clarke JD , Saravanan P , Voigt N , Dobrev D , Eisner DA , Trafford AW & Dibb KM (2011). Transverse tubules are a common feature in large mammalian atrial myocytes including human. Am J Physiol Heart Circ Physiol 301, H1996–H2005. 21841013 10.1152/ajpheart.00284.2011 PMC3213978

[tjp12537-bib-0046] Roca TP , Pigott JD , Clarkson CW & Crumb WJ, Jr. (1996). L‐type calcium current in pediatric and adult human atrial myocytes: evidence for developmental changes in channel inactivation. Pediatr Res 40, 462–468. 8865285 10.1203/00006450-199609000-00016

[tjp12537-bib-0047] Rockwood K , Blodgett JM , Theou O , Sun MH , Feridooni HA , Mitnitski A , Rose RA , Godin J , Gregson E & Howlett SE (2017). A frailty index based on deficit accumulation quantifies mortality risk in humans and in mice. Sci Rep 7, 43068. 28220898 10.1038/srep43068 PMC5318852

[tjp12537-bib-0048] Sankaranarayanan R , Li Y , Greensmith DJ , Eisner DA & Venetucci L (2016). Biphasic decay of the Ca transient results from increased sarcoplasmic reticulum Ca leak. J Physiol 594, 611–623. 26537441 10.1113/JP271473 PMC4785612

[tjp12537-bib-0049] Satoh H , Delbridge LMD , Blatter LA & Bers DM (1996). Surface:Volume relationship in cardiac myocytes studied with confocal microscopy and membrane capacitance measurements: Species dependence and developmental effects. Biophys J 70, 1494–1504. 8785306 10.1016/S0006-3495(96)79711-4 PMC1225076

[tjp12537-bib-0050] Schmidt U , del MF , Miyamoto MI , Matsui T , Gwathmey JK , Rosenzweig A & Hajjar RJ (2000). Restoration of diastolic function in senescent rat hearts through adenoviral gene transfer of sarcoplasmic reticulum Ca(2+)‐ATPase. Circulation 101, 790–796. 10683354 10.1161/01.cir.101.7.790

[tjp12537-bib-0051] Tipparaju SM , Kumar R , Wang Y , Joyner RW & Wagner MB (2004). Developmental differences in L‐type calcium current of human atrial myocytes. Am J Physiol Heart Circ Physiol 286, H1963–H1969. 14715512 10.1152/ajpheart.01011.2003

[tjp12537-bib-0052] Trafford AW , Díaz ME & Eisner DA (1999). A novel, rapid and reversible method to measure Ca buffering and timecourse of total sarcoplasmic reticulum Ca content in cardiac ventricular myocytes. Pflügers Archiv 437, 501–503. 9914410 10.1007/s004240050808

[tjp12537-bib-0053] Trafford AW , Díaz ME & Eisner DA (2001). Coordinated control of cell Ca^2+^ loading and triggered release from the sarcoplasmic reticulum underlies the rapid inotropic response to increased L‐type Ca^2+^ current. Circ Res 88, 195–201. 11157672 10.1161/01.res.88.2.195

[tjp12537-bib-0054] Trafford AW , Díaz ME , Negretti N & Eisner DA (1997). Enhanced Ca^2+^ current and decreased Ca^2+^ efflux restore sarcoplasmic reticulum Ca^2+^ content following depletion. Circ Res 81, 477–484. 9314828 10.1161/01.res.81.4.477

[tjp12537-bib-0055] Tsang TS , Abhayaratna WP , Barnes ME , Miyasaka Y , Gersh BJ , Bailey KR , Cha SS & Seward JB (2006). Prediction of cardiovascular outcomes with left atrial size: is volume superior to area or diameter? J Am Coll Cardiol 47, 1018–1023. 16516087 10.1016/j.jacc.2005.08.077

[tjp12537-bib-0056] Varro A , Negretti N , Hester SB & Eisner DA (1993). An estimate of the calcium content of the sarcoplasmic reticulum in rat ventricular myocytes. Pflügers Archiv 423, 158–160. 8488088 10.1007/BF00374975

[tjp12537-bib-0057] Vaziri SM , Larson MG , Benjamin EJ & Levy D (1994). Echocardiographic predictors of nonrheumatic atrial fibrillation. The Framingham Heart Study. Circulation 89, 724–730. 8313561 10.1161/01.cir.89.2.724

[tjp12537-bib-0058] Venetucci L , Trafford AW & Eisner DA (2007). Increasing ryanodine receptor open probability alone does not produce arrhythmogenic Ca^2+^ waves: threshold Ca^2+^ content is required. Circ Res 100, 105–111. 17110597 10.1161/01.RES.0000252828.17939.00

[tjp12537-bib-0059] Voigt N , Heijman J , Wang Q , Chiang DY , Li N , Karck M , Wehrens XH , Nattel S & Dobrev D (2014). Cellular and molecular mechanisms of atrial arrhythmogenesis in patients with paroxysmal atrial fibrillation. Circulation 129, 145–156. 24249718 10.1161/CIRCULATIONAHA.113.006641 PMC4342412

[tjp12537-bib-0060] Voigt N , Li N , Wang Q , Wang W , Trafford AW , Abu‐Taha I , Sun Q , Wieland T , Ravens U , Nattel S , Wehrens XH & Dobrev D (2012). Enhanced sarcoplasmic reticulum Ca^2+^ leak and increased Na^+^‐Ca^2+^ exchanger function underlie delayed afterdepolarizations in patients with chronic atrial fibrillation. Circulation 125, 2059–2070. 22456474 10.1161/CIRCULATIONAHA.111.067306 PMC4663993

[tjp12537-bib-0061] Voigt N , Pearman CM , Dobrev D & Dibb KM (2015). Methods for isolating atrial cells from large mammals and humans. J Mol Cell Cardiol 86, 187–198. 26186893 10.1016/j.yjmcc.2015.07.006

[tjp12537-bib-0062] Walden AP , Dibb KM & Trafford AW (2009). Differences in intracellular calcium homeostasis between atrial and ventricular myocytes. J Mol Cell Cardiol 46, 463–473. 19059414 10.1016/j.yjmcc.2008.11.003

[tjp12537-bib-0063] Wongcharoen W , Chen YC , Chen YJ , Chen SY , Yeh HI , Lin CI & Chen SA (2007). Aging increases pulmonary veins arrhythmogenesis and susceptibility to calcium regulation agents. Heart Rhythm 4, 1338–1349. 17905341 10.1016/j.hrthm.2007.06.023

[tjp12537-bib-0064] Xu GJ , Gan TY , Tang BP , Chen ZH , Jiang T , Song JG , Guo X & Li JX (2013). Age‐related changes in cellular electrophysiology and calcium handling for atrial fibrillation. J Cell Mol Med 17, 1109–1118. 23837844 10.1111/jcmm.12084 PMC4118170

[tjp12537-bib-0065] Yeh YH , Wakili R , Qi X , Chartier D , Boknik P , Kaab S , Ravens U , Coutu P , Dobrev D & Nattel S (2008). Calcium handling abnormalities underlying atrial arrhythmogenesis and contractile dysfunction in dogs with congestive heart failure. Circ Arrhythmia Electrophysiol 1, 93–102. 10.1161/CIRCEP.107.754788 19808399

